# Use of porous trabecular metal augments with impaction bone grafting in management of acetabular bone loss: Early to medium-term results

**DOI:** 10.3109/17453674.2012.718518

**Published:** 2012-08-25

**Authors:** W Steven Borland, Raj Bhattacharya, James P Holland, Nigel T Brewster

**Affiliations:** Department of Trauma and Orthopaedics, Freeman Hospital, High Heaton, Newcastle upon Tyne, UK

## Abstract

**Background:**

The use of impaction grafting in revisions with larger acetabular bone defects has mixed outcomes and sometimes high failures rates.

**Patients and methods:**

This prospective, single-center study involved a consecutive series of 24 patients who underwent complex reconstruction of the acetabulum using a trabecular metal augment, impaction bone grafting, and a cemented high-density polyethylene cup. Patients were followed for median 5 (3–7) years.

**Results:**

The 2-year WOMAC pain, function, and stiffness scores improved, as did certain components (bodily pain, physical function, role physical, role emotional, physical component score, and social function) of the SF-36 (p < 0.05). 23 of the patients were very satisfied with the overall outcome of the surgery and would have undergone the surgery again for a similar problem, and 19 reported great improvement in their quality of life after surgery. Radiographs at the latest follow-up revealed incorporation of the augment with mean change in acetabular component inclination of less than 1 degree (p > 0.05) and cup migration of less than 5 mm in both horizontal and vertical axes (p > 0.05). 1 patient required further revision at 13 months and was found to have a fractured augment at re-revision.

**Interpretation:**

This study shows that trabecular metal augments are effective in filling the bone defect and provide a stable foundation for impaction bone grafting. We found satisfactory clinical and radiographic results using this technique, with low failure rate at a median follow-up time of 5 years.

While revision of the acetabular component with minimal bone loss can be straightforward, one can often encounter substantial bone loss which makes reconstruction difficult. Various surgical options including impaction grafting (Schreurs et al. 2001), reconstruction cages (Berry et al. 1999, Gross 1999, Saleh et al. 2000, Gross and Goodman 2004), bi-lobed acetabular components (DeBoer and Christie 1998, Berry et al. 2000, Chen et al. 2000) and structural allograft (Paprosky et al. 1994, Dewal et al. 2003) are available to deal with bone loss. Trabecular metal (TM) augments are a more recently available option to address bone loss and restore the center of the hip.

TM is a biomaterial made of porous tantalum that has a 3D structure and is highly porous (Figure 1). TM has a porosity similar to that of cancellous bone, encouraging ingrowth (Bobyn et al. 1980, Cohen 2002, Unger et al. 2005). The elastic modulus of TM is more similar to that of cancellous and cortical bone than is the elastic modulus of other commonly used metals (O’Keefe et al. 1999). The coefficient of friction against bone is higher than other porous coatings such as sintered beads and fibers.

**Figure 1.**
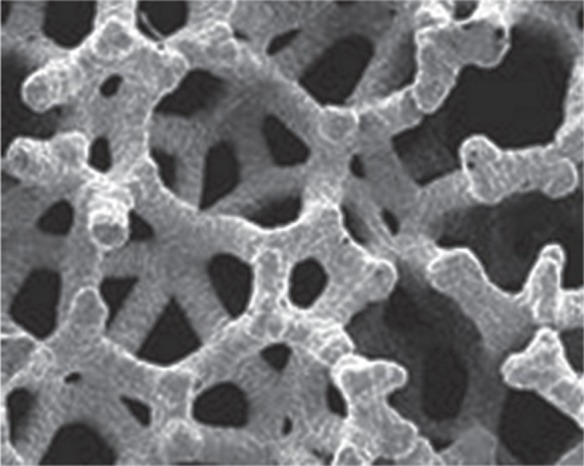
Microscopic structure of trabecular metal

The high coefficient of friction increases the grip on the bone, and may be more effective in preventing early failure than other structures that have been used in the past as a support for impaction bone grafting.

Impaction grafting of acetabular defects in conjunction with meshes has been shown to have mixed outcomes: 12% failure at 10 years (van Haaren et al. 2007) and 28% failure at 7 years (van Egmond et al. 2011). As TM augments are very stable mechanically, we hypothesized that using an augment to fill a defect first and then to perform impaction grafting on top would provide more stability to the subsequently cemented cup than a mesh and bone graft would.

We report our experience with TM augments to improve stabilization of impaction grafts and subsequently cemented cups in revision cases in a tertiary referral practice.

## Patients and methods

This study involved a consecutive series of patients who underwent acetabular reconstruction using TM augments (Zimmer Inc., Warsaw, IN) as a support for impaction bone grafting and cemented acetabular cups from February 2004 through March 2008. The follow-up period was at least 2 years. We present all the cases in which this technique was used in our department, including those during the period with the learning curve. Independently trained research assistants collected the data prospectively.

At our unit, during the inclusion period 70 hip revision procedures were performed every year, giving 280 procedures over the 4 years. Two-thirds of these procedures were performed for acetabular problems. In the 180 acetabular revision procedures performed in the time period, 24 patients (13 men) met the criteria of massive aseptic acetabular bone loss to warrant this technique. The mean age was 62 (24–87) years. The patients had undergone between 1 and 4 previous surgeries before the procedure.

Patients were assessed 6 weeks or less before surgery and at 12 weeks and 1 year. They were assessed annually thereafter. Median follow-up was 61 (32–81) months. None of the patients were lost to follow-up, but the 2-year postoperative questionnaire was completed by 21 of the 24 patients.

All the patients were operated by or under the direct supervision of the senior authors (NTB and JPH). A posterior approach was used in all cases. The existing cup was explanted, and the interface membrane removed and acetabulum cleaned. The acetabular defect was then quantified according to Paprosky grading (Paprosky et al. 1994) and recorded. There were 15 Paprosky grade 3A defects and 9 grade 3B defects.

**Figure 2.**
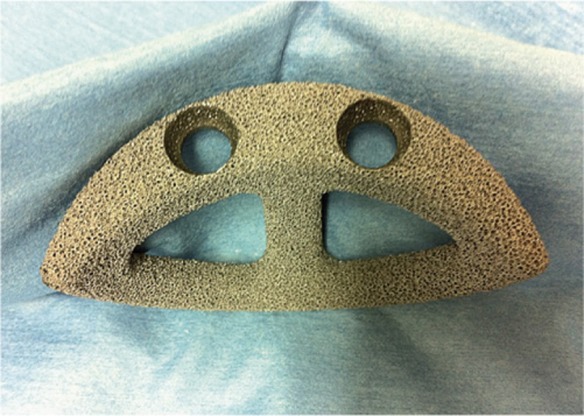
Wedge type trabecular metal augment.

Sequential gentle acetabular reaming was carried out to identify the best fit. The TM augments are available in different sizes and shapes, making it easier for the surgeon to choose the correct size of implant. The appropriate size and number of augments were then chosen for the defect (Figure 2).

The augment was tested with the trial cup to ensure best cover and support. It was secured with one or two 6.5-mm screws. In all but 1 case, a single wedge-shaped augment was used. In that particular case, a wedge-shaped augment was used in conjunction with a disc-shaped augment for a medial wall defect.

Morsellised allograft from donor femoral head was then packed around and over the augment and secured in place using an appropriate-sized acetabular reamer in reverse. A low-profile Exeter cup (Stryker, Berkshire, UK) was selected to give at least 2–3 mm of cement mantle. Cement was inserted on top of the morsellised graft and pressurized. The Exeter cup was then cemented in place on top of the impacted graft. Femoral revision was then carried out as required in 7 of the 24 patients. Antibiotics and thromboprophylaxis were prescribed as per out unit protocol.

Patients were mobilized with partial weight bearing with crutches for 6 weeks, followed by transition to full weight bearing as tolerated over the following 6 weeks. By 3 months, all patients were allowed full weight bearing.

Patients completed questionnaires preoperatively and at each postoperative assessment. Hip status was assessed using the Western Ontario and McMaster Universities’ (WOMAC) questionnaire (Theiler et al. 2002) Scores for pain, function, and stiffness were transformed to a 0- to 100-point scale, with 0 indicating extreme pain/functional disability/stiffness and 100 indicating no pain/functional disability/stiffness. This method has been widely reported (Wright et al. 2004, Wylde et al. 2008, Lingard et al. 2009). Generic health was assessed using the SF-36 version 2 health survey (Ware 2000) with its 10 components ranging from 0 to 100 with 100 being best. Postoperatively, satisfaction data were recorded using a validated measure of satisfaction (Mahomed et al. 1998). This questionnaire includes 4 questions asking about satisfaction with the overall outcome, pain relief, ability to perform activities of daily living, and ability to participate in leisure activities. Responses are given on a 4-point Likert scale, which ranges from very satisfied to very dissatisfied. In addition to validated questions, the patients were also asked whether they would undergo the operation again and how much the surgery had improved their quality of life. The responses were given on a 5-point Likert scale that ranged from “a great improvement” to “the quality of my life is worse”.

### Radiographic analysis

**Figure 3.**
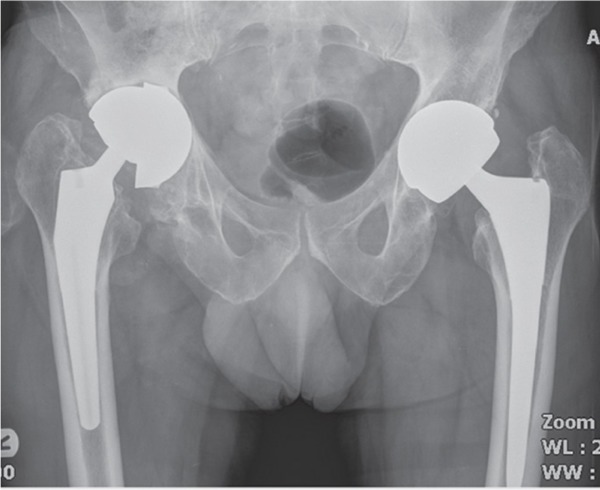
Preoperative failed THR with acetabular bone loss.

**Figure 4.**
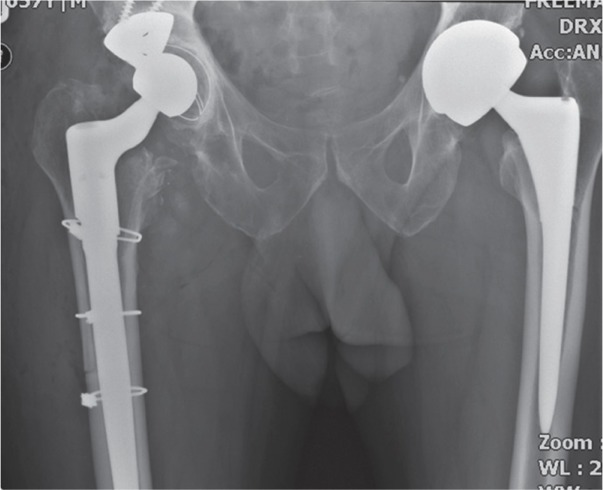
Postoperative reconstruction film.

Standard AP pelvis and lateral hip radiographs were obtained. The radiographs were all digital images and each radiograph was calibrated for measurement using the head size of the prosthesis. Preoperative radiographs, radiographs taken immediately postoperatively, and the most recent follow-up radiographs were analyzed (Figures 3 and 4).

The preoperative radiographic analysis included classification of bone defects using the Paprosky classification, measurement of the abduction angle of the existing cup (in the case of revisions), and the position of the existing cup (in the case of revisions). Teardrop sign is a reliable and a constant feature seen on AP radiographs of the pelvis. We used this key landmark to determine the position of the cup. A horizontal line was marked along the inferior border of the 2 teardrops on an AP pelvic radiograph, a vertical line bisecting the ipsilateral teardrop was then marked, and the point of intersection was noted. The horizontal (x-) and vertical (y-) distance from this intersection point and the most inferior point of the rim of the cup gives the position of the cup in relation to the teardrop. For the purposes of this study, we assumed that the ideal cup position is where the inferior most point of the rim of the cup lies adjacent to the teardrop (i.e. the vertical and horizontal distance are 0 mm). This assumption was made on the basis of this being the anatomical position of the cup within the acetabulum.

The postoperative radiographic analyses included measurement of the abduction angle and acetabular component position using the methodology described above. The most recent follow-up radiographs were also assessed for (1) evidence of bony ingrowth or lucency around the TM augments or disc and (2) quality of the cement mantle (i.e. evidence of cup loosening from comparative radiographs). Migration of the acetabular component was determined by calculating the difference between the cup position (x- and y-axes) at the most recent follow-up and the radiograph taken immediately postoperatively.

### Statistics

In order to determine whether the data followed a normal distribution, the Kolmogorov-Smirnov test was used. If p < 0.05, non-parametric tests rather than parametric tests were used. To compare continuous variables, paired t-tests were used for parametric data and the Mann-Whitney U test was used for variables not having a Gaussian distribution. All the tests were 2-tailed and the 5% significance level was used throughout. The chi-square test was used to compare categorical data. Statistical analyses were performed using SPSS software version 11.

### Ethics

The patients were not entered into a clinical trial; no formal ethics approval was therefore required. All procedures were performed by a trained consultant orthopaedic surgeon.

## Results

### Clinical outcome

**Figure 5.**
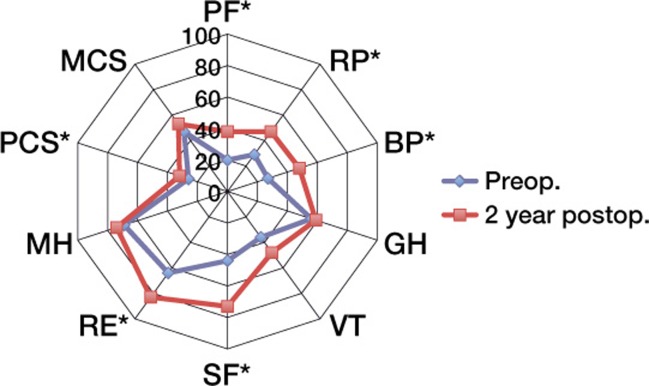
Radar graph of SF-36 score. Scores with * showed a statistically significant difference (p < 0.05). PF* Physical function RP* Role physical BP* Bodily pain GH Generic health VT Vitality SF* Social functioning RE* Role emotional MH Mental health PCS* Physical component score MCS Mental component score

WOMAC pain, function, and stiffness scores had improved at 2 years (p < 0.001) (Table 1). Certain components of the SF-36 score (bodily pain, physical function, role physical, role emotional, physical component, and social function) had also improved at 2 years (p < 0.05) (Figure 5).

**Table 1.** WOMAC scores

Outcome measure	Preoperative score median (range)	Postoperative (2-year) score median (range)	p-value
WOMAC			
Pain	38 (10–85)	90 (40–100)	< 0.001
Function	38 (1–81)	77 (40–100)	< 0.001
Stiffness	38 (0–88)	81 (25–100)	< 0.001

23 patients were very satisfied with the overall outcome of the surgery. 23 would have had the surgery again for a similar problem, if indicated, and 19 experienced a great improvement in their quality of life after surgery.

### Radiographic outcome

Radiographs taken immediately postoperatively showed an improvement in the abduction angle (p = 0.97) and an improvement in the cup position (p < 0.001) (Table 2).

**Table 2.** Difference between preoperative and immediately postoperative cup position

	Preoperatively	Immediately postoperatively	p-value
Abduction angle (degrees)	55 (1–107)	46 (31–56)	0
Cup position (mm)			
x-axis	16 (7–35)	7 (0–25)	< 0.001
y-axis	18 (3–41)	9 (0–21)	< 0.001

No significant difference was found between abduction angle in the immediate postoperative period and in the most recent follow-up radiographs (p = 0.187). Mean cup migration at last follow-up was less than 5 mm in both horizontal (p = 0.138) and vertical axes (p = 0.591) (Table 3).

**Table 3.** Change of cup position at the last follow-up

	Difference^a^	p-value
Change of abduction angle (degrees)	< 1	0.187
Cup migration (mm)		
x-axis	< 5	0.138
y-axis	< 5	0.591

**^a^**Difference between the immediately postoperative follow-up and the most recent follow-up

In 5 cases, the migration was more than 5 mm on each axis. 1 patient had a fractured augment and required further revision 13 months after his revision surgery. He had presented with severe hip pain after a fourth revision surgery that was reconstructed using this technique. No other patients required revision.

3 patients had Delee-Charnley zone 2-cup lucency at the bone-cement interface, but this was not progressive. 2 patients had lucency in zones 2 and 3 at the bone-cement interface at the latest follow-up, indicating a potentially loose cup, but they were both asymptomatic. No evidence of loosening was found around the augments or the screws securing the augments. The patient who had an additional medial wall TM disc showed good radiographic incorporation into the surrounding bone.

### Complications

There was no dislocation or deep infection. 1 patient reported paresthesia along the sciatic nerve distribution without motor weakness, and this partially recovered.

## Discussion

Management of acetabular defects can be challenging. Various reconstructions have been tried, with varying degrees of success. Uncemented jumbo sockets are widely used (Dearborn and Harris 2000, Whaley et al. 2001, Patel et al. 2003). These allow direct contact between the implant and the host bone. However, no attempt is made to restore the lost bone stock. This may require the cup to be placed in a “high hip center”. This interferes with the mechanics of the hip joint (Bozic et al. 2004), does not restore bone loss, and can lead to early loosening. An oblong or bi-lobed socket can theoretically restore the hip center and gain immediate host bone contact, but high early failure rates have been shown (Chen et al. 2000). Impaction bone grafting involves containment of bony defects and filling them with compacted morsellised bone graft, and cementing a socket into the graft. Acceptable medium-term results are achieved with this procedure (Schmalzried et al. 1992, Berry et al. 1999, Gross 1999, Saleh et al. 2000, Gross and Goodman 2004). There is concern regarding subsidence and early failure after grafting of large segmental defects. Structural allograft can be used to replace lost bone stock, but there is poor host-graft incorporation and also high infection rates (Paprosky et al. 1994, Dewal et al. 2003).

The outcomes of the use of impaction bone grafting in conjunction with mesh vary. Reports have shown a failure rate of up to 28% after 7 years (van Haaren et al. 2007). It should be stressed that while our short-term results are good, the long-term results may be no better than with using a mesh.

The combination of augments and cemented cups may also be attractive when considering potential failure. Failing uncemented metal-backed cups will result in friction and wear between the augment and the cup, thus generating a lot of wear from metal debris. This is probably not the case when there is failure with cemented cups.

We used TM augments to restore segmental bony defects before using a compact morsellised bone grafting technique and a cemented socket. This technique allowed us to place the augment in the required alignment for the defect, as it did not determine the final alignment of the socket. We obtained satisfactory short-term results, both clinically and radiographically. The sequential radiographs showed no statistically significant changes in cup inclination and migration over the postoperative period. This indicates that the TMT augment provides a good bed for incorporation of the bone graft into the acetabular bone and the augment.

Re-revision was required in 1 of 24 hips because of severe pain after a fractured augment, 13 months after the revision surgery. We believe that the failure occurred because of the stress going through the augment, accentuated by a relatively open (high-abduction-angle) position of the acetabular component. We have since modified our technique, with a deliberate attempt to keep the acetabular component relatively closed.

The use of TM augments is gaining popularity in the management of bone loss in acetabular reconstruction. The TM augment is used to fill the defect and is packed with morsellised bone graft. The reconstruction is then completed using a cementless cup or a cemented cup. In a study using TM augments and cementless cup, Nehme et al. (2004) reported good early to medium-term results. Similarly, in a series of 28 hips with a Paprosky type IIIA defect and 13 hips with a type IIIB defect, Sporer and Paprosky (2006) found no failures after aseptic loosening. Weeden and Schmidt (2007) also reported no revisions for aseptic loosening when reviewing 43 Paprosky type IIIA or type IIIB acetabular revisions at a mean follow-up time of 3 years. Lakstein et al. (2009) found an 8% failure rate at 4 years on average when TM cups (Zimmer) without augments were used in patients with < 50% host bone contact.

While other centers have used this technique, to our knowledge this is the largest cohort with the longest follow-up. We continue to monitor these patients, and a larger series with longer follow-up will be required to determine the long-term outcome of these augments.
